# Recurrent Implantation Failure May Be Identified by a Combination of Diagnostic Biomarkers: An Analysis of Peripheral Blood Lymphocyte Subsets

**DOI:** 10.3389/fendo.2022.865807

**Published:** 2022-07-22

**Authors:** Jun-Ying Cai, Yuan-Yuan Tang, Xi-He Deng, Yan-Juan Li, Gui Liang, Ya-Qing Meng, Hong Zhou

**Affiliations:** Department of Reproductive Center, Maternal and Child Health Hospital and Obstetrics and Gynecology Hospital of Guangxi Zhuang Autonomous Region, Nanning, China

**Keywords:** peripheral blood lymphocyte subsets, T cells, natural killer cells, diagnostic biomarker, flow cytometry, recurrent implantation failure

## Abstract

**Background:**

Recurrent implantation failure (RIF) is a challenge during assisted reproductive technology (ART). In the present study, potential diagnostic biomarkers for the immune status of peripheral blood lymphocyte subsets in patients with RIF were analyzed, with the aim of identifying novel biomarkers that may predict RIF.

**Methods:**

A total of 41 participants, including 21 women with RIF and 20 fertile controls, were included in the present study. Functional analysis was performed and the cytokine status of natural killer (NK), T, CD8+ T, T helper (Th), and γδ T cells which are lymphocyte subsets in peripheral blood was measured using flow cytometry. Binary logistic regression analysis adjusted for T follicular helper 1 (Tfh1), Tfh2, Tfh17, and early NK cells was performed to determine the relationship between the peripheral blood lymphocyte subsets and RIF. Potential diagnostic biomarkers were assessed by logistic regression analysis and receiver operating characteristic curves.

**Results:**

There were significantly more Tfh1, Tfh17, and NK cells in the RIF group compared with the control group (all *P* < 0.05). However, the percentage of T, regulatory T (Tregs), and Tfh2 cells, as well as early inhibitory NK cells, was significantly lower in the RIF group compared with the control group (all *P* < 0.05). Following logistics regression analysis, Treg, Tfh17, and early inhibitory NK cells exhibited significant differences between the two groups. Combination diagnosis using these 3 biomarkers had a higher area under the curve of 0.900 (95% confidence interval: 0.808–0.992, *P* < 0.001) in the RIF group compared with that in the control group.

**Conclusion:**

T, Tregs, Tfh1, Tfh2, Tfh17, NK cells, and early inhibitory NK cells may play important regulatory roles in embryo implantation. The combination of 3 molecular markers (Treg, Tfh17, and early inhibitory NK cells) could provide a high diagnostic value for women with RIF, thus providing novel potential biomarkers for RIF in ART. The present findings could provide a reference either for the clinical treatment of patients with RIF or for future large, well-designed studies.

## Introduction

Recent advances in the optimization of assisted reproductive technology (ART) have led to marked improvements in embryo implantation. However, low implantation rates remain a challenge. *In vitro* fertilization (IVF) is associated with low pregnancy rates due to recurrent implantation failure (RIF), which has become a research hotspot in ART. RIF, which can lead to considerable financial losses, as well as inflict physical or mental pressures on patients and their families, has been reported to have an incidence rate of 5–10% in women undergoing IVF cycles and therefore requires urgent attention (1). However, no unified and standardized diagnostic methods have been reported for RIF worldwide.

Embryo implantation mainly includes three stages: apposition, adhesion, and invasion (2). RIF may be caused by multiple factors, including parental chromosomal abnormalities, embryo quality, endometrial receptivity, and immunological disturbance (3). Among them, immunologic factors are thought to play an important role in RIF. At the maternal–fetal interface, a multitude of immune cells, including T cells, natural killer (NK) cells, macrophages, and dendritic cells, form a vast network of cellular connections (4). A cellular immunological abnormality in any of these cell types may lead to pregnancy failure. Currently, the etiology of RIF is unclear. Considerable evidence suggests that RIF is caused as a result of maternal immune activation in semi-allograft embryos that will be rejected by the mothers’ endometrium (5, 6).

NK cells are a type of lymphocyte found in human peripheral blood and the endometrium; they are primarily responsible for nonspecific immunity. NK cells can recognize target cells through natural cytotoxicity receptors (7). Approximately 90% of peripheral blood NK cells turn into cytotoxic NK cells (4). Compared with NK cells, T cells, which take up 10–20% of the lymphocytes in the decidua, are responsible for cellular immunity. A total of 30–45% of the T cells are CD4^+^ and 45–75% are CD8^+^ (8). A relationship between T cell activation and Th1 immunity was reported in women with RIF and recurrent pregnancy losses (RPLs) (9). The findings by Yin et al. indicated that peripheral CD8^+^ T cells may contribute to immune disorders in women with RIF (10).

In recent years, the detection of peripheral blood lymphocyte subsets has been used for the diagnoses of other diseases, including diffuse large B-cell lymphomas and coronavirus disease 2019 (COVID-19) (11, 12). The diagnostic value for women with RIF remains unknown. In the present study, a comprehensive analysis was conducted on the subsets of peripheral blood lymphocytes, including T cells, NK cells, and γδ T cells, in patients with RIF, as compared with the same subsets in patients with successful pregnancies by embryo transfer. The aim of the present study was to explore the regulatory mechanisms of peripheral blood lymphocytes in patients with RIF during the implantation window and attempt to find novel and valuable diagnostic biomarkers for RIF in ART.

## Materials and Methods

### Study Population

The present study was carried out on patients from the Reproductive Medicine Center, Maternal and Child Health Hospital of Guangxi Zhuang Autonomous Region and approved by the Ethics Committee of the Maternal and Child Health Hospital of Guangxi Zhuang Autonomous Region for Reproductive Medicine. Informed consent was obtained from all individual participants.

Participants were recruited between January and December 2018 according to the following inclusion criteria: (i) age or <35 years; (ii) normal menstrual cycle of 21–35 days; (iii) single frozen-thawed blastocyst transfer cycle; and (iv) embryo grade of ≥3BB, according to the Garden and Lane criteria (13). Patients with one or more than one of the following conditions were excluded: uterine adenomyosis, endometriosis, intrauterine occupational disease, intrauterine adhesions, an endocrine disorder, abnormal chromosome, genital tract malformation, and inflammation. In the end, a total of 41 participants were selected and divided into two groups: the RIF and control groups. The participants who experienced pregnancy failure after at least 3 consecutive IVF attempts (involving either fresh or frozen-thawed cycles) and transplantation of 1–2 embryos of high-grade quality in each cycle were included in the RIF group (14). Participants with fallopian tube malfunctions who were able to achieve successful IVF-assisted pregnancies and their babies (aged >1 years) were included in the control group. The information collected from each participant included age, body mass index (BMI), infertility time, endometrial thickness, and basal and mid-luteal period (luteinizing hormone day 5–7) sex hormone levels.

### Sample Collection and Flow Cytometry

Peripheral blood samples were collected during the mid-luteal period. Blood samples (100 μl) and a mixture of antibodies for CD4^+^ T, CD8^+^ T, NK, γδT, Th, and B cells (10 μl) were added to six tubes, respectively, according to the manufacturers’ instructions. The color scheme of antibodies and the combination of surface antibodies for each cell are detailed in **Supplementary Tables S1**, **S2**. An additional step-wise gating procedure for the flow cytometry can be seen in **Supplementary Figure S1**, including the gate for CD4^+^ T, CD8^+^ T, NK, and γδ cells and their subsets. Following shaking for 30 s, cells were incubated at room temperature to avoid light for 15 min. Cells were then lysed and fixed with 800 μl AKC lysing solution (Becton, Dickinson and Company) in an incubator in the dark at 4°C for 15 min, followed by centrifugation at 1,000×*g* for 5 min. After removing the supernatant, 200 μl AKC lysing solution was added to each tube while avoiding the light, followed by further incubation for 3 min after which the pellets were washed with 2 ml PBS and centrifuged at 1,000×*g* for 5 min. Finally, 350 μl PBS was added to each tube and centrifuged, and the supernatants were removed. The pellets were measured in a FACSCanto flow cytometer (Mountain View; Becton, Dickinson and Company). The results were analyzed using FlowJo software (TreeStar).

### Statistical Analysis

All statistical analysis was performed with SPSS (version 23.0; IBM Corp.). The Kolmogorov–Smirnov test was used to confirm whether the data were normally distributed. All data are presented as the mean ± standard deviation or a median (25–75% quartiles). Differences between two groups were compared using a Student’s *t* test or Mann–Whitney *U* tests. Considering that T and NK cells are large group of lymphocytes, their fluctuation and significance are markedly affected by cell changes in each of the other lymphocyte subsets. Therefore, binary logistic regression analysis adjusted for the 5 indicators was performed to determine differences among them, and an ROC curve was created. Logistic regression analysis and areas under the curve (AUC) of ROC curves with 95% CI were used to predict diagnostic value. The statistical tests were two-tailed and *P* < 0.05 was considered to indicate a statistically significant difference.

## Results

### Baseline Characteristics

There were 21 patients in the RIF group and 20 in the control group. The median age of participants in the RIF group and fertile controls were 32.00 (28.50–32.50) and 30.00 (28.00–31.50) years, respectively. The characteristics of the two groups are presented in **Table 1**. There was no significant difference in age, BMI, infertility time, baseline hormone levels of estradiol (E2) and progesterone (P), mid-luteal period of endometrial thickness, E2, P, and luteinizing hormone levels between the two groups (all *P* > 0.05).

**Table 1:** Baseline characteristics of the study population.

Variables	RIF group (*n* = 21)	Control group (*n* = 20)	*P* value
Age (years)	32 (28.5–32.5)	30 (28–31.5)	0.247
Body mass index (kg/m^2^)	20.81 (19.8–22.04)	21.08 (20.26–22.83)	0.486
Infertility time (years)	3 (1.5–5.5)	2 (1–3)	0.083
Baseline sex hormone level			
E2 (pmol/l)	165.8 (129–213.25)	137.3 (117.2–177.55)	0.170
P (nmol/l)	1.59 ± 0.54	1.7 ± 0.68	0.597
Mid-luteal period			
Endometrial thickness (mm)	9.19 ± 2.22	9.90 ± 1.77	0.267
LH (mIU/ml)	35.25 ± 18.46	34.25 ± 21.15	0.874
E2 (pmol/l)	1,309.15 ± 475.23	1,250.23 ± 664.19	0.747
P (nmol/l)	2.33 ± 1.07	2.06 ± 1.15	0.449

RIF, recurrent implantation failure; E2, estradiol; P, progesterone; LH, luteinizing hormone. Data are presented as the mean ± standard deviation or a median (25–75% quartiles). The P value was calculated using a Student’s t test or Mann–Whitney U test.

### Comparisons of Lymphocyte Subsets Between Groups

To investigate the potential relationship between the immune status of peripheral blood lymphocytes and RIF, the lymphocyte subsets were first assessed between the two groups. Results showed that patients with RIF had a significantly lower percentage of T cells (*P* = 0.010) and a significantly higher percentage of NK cells (*P* = 0.019) in their peripheral blood samples (**Table 2**). No significant differences were observed in the other lymphocyte subsets (T helper, killer T cells, and double-positive T lymphocytes) or in the T helper (Th) to T cytotoxic (Tc) ratio, between the two groups (**Table 2**).

**Table 2:** Comparisons of lymphocyte subsets between the recurrent implantation failure and control groups.

Cell type	RIF group (*n* = 21)	Control group (*n* = 20)	*P* value
T cells (% of lymphocyte)	64.57 ± 10.22	72.81 ± 9.17	0.010
Natural killer cells (% of lymphocyte)	20.27 ± 9.95	13.63 ± 7.13	0.019
Th cells (% of T cells)	54.65 ± 9.04	58.73 ± 8.41	0.143
Killer T cells (% of T cells)	38.20 (31.05, 45.00)	35.75 (27.58, 41.88)	0.235
Double positive T lymphocytes (% of T cells)	1.50 (0.84, 2.28)	1.52 (1.01, 1.98)	0.725
Th/T cytotoxic	1.55 (1.05, 1.77)	1.63 (1.21, 2.48)	0.24

RIF, Recurrent implantation failure; Th, T helper. The data are presented as the mean ± standard deviation or a median (25–75% quartiles). A Student’s t test or Mann–Whitney U test was conducted. P < 0.05 was considered to indicate a statistically significant difference.

### Comparisons of Functions and Differentiation of T Cell Subsets Between Groups

To assess the functions and differentiation of T cells between groups, an analysis of T cells in peripheral blood samples was performed (**Table 3**). The percentage of CD3^+^ regulatory T cells (Tregs) in the RIF group was found to be significantly lower than that in the control group (*P* = 0.005). No significant differences were observed in the other subsets. As compared with the control group, the RIF group exhibited a higher percentage of Tfh1 (*P* = 0.024) and Tfh17 (*P* = 0.004) cells and a lower percentage of Tfh2 cells (*P* = 0.008) among the total number of Tfh cells. In addition, the RIF group exhibited a significantly higher percentage of Th17 to Th2 (*P* = 0.003) and Th1^+^Th17 to Th2 (*P* = 0.002). No differences were observed in the functions of CD8^+^ T and γδ T cell subsets between the two groups.

**Table 3:** Comparisons of functions and differentiation of T cell subsets between the recurrent implantation failure and control groups in peripheral blood samples.

Cell type	RIF group (*n* = 21)	Control group (*n* = 20)	*P* value
T cell functional subsets			
Naïve CD4^+^ T cells (% of CD4 T cells)	19.65 ± 16.22	22.03 ± 10.26	0.581
Terminal differentiated CD4^+^ T cells (% of CD4 T cells)	27.22 ± 12.82	25.09 ± 11.87	0.584
Central memory CD4^+^ T cells (% of T cells)	5.48 (2.66, 8.30)	5.54 (3.84, 8.73)	0.322
Effective memory CD4^+^ T cells (% of CD4 T cells)	47.60 ± 13.10	48.23 ± 12.21	0.774
Exhaustion CD4^+^ T cells (% of CD4 T cells)	2.00 (0.48, 4.58)	2.23 (0.30, 5.59)	0.794
Functional CD4^+^ T cells (% of CD4 T cells)	98.00 (95.40, 99.55)	97.80 (94.40, 99.68)	0.834
Tregs (% of CD3 T cells)	2.24 ± 0.91	3.17 ± 1.09	0.005
Naïve CD8^+^ T cells (% of CD8 T cells)	21.84 ± 9.05	25.58 ± 14.70	0.336
Terminal differentiation CD8^+^ T cells	51.50 ± 13.44	43.93 ± 12.15	0.066
Central memory CD8^+^ T cells (% of CD8 T cells)	0.32 (0.22, 0.5)	0.36 (0.24, 0.75)	0.498
Effective memory CD8^+^ T cells (% of CD8 T cells)	20.00 (15.50, 41.15)	27.60 (21.23, 36.75)	0.285
Exhaustion of CD8^+^ T cells (% of CD8 T cells)	10.4 (1.94, 31.00)	13.10 (4.28, 32.28)	0.368
Inactive specificity CD8^+^ T cells (% of effective memory CD8^+^ T cells)	64.74 ± 15.85	57.78 ± 22.08	0.252
Inactive specificity terminal differentiation CD8^+^ T cells (% of terminal differentiation CD8^+^ T cells)	57.00 ± 20.33	53.22 ± 22.99	0.579
Persistent viral specificity CD8^+^ T cells (% of effective memory CD8^+^ T cells)	35.25 ± 15.86	42,.21 ± 22.08	0.251
Persistent viral specificity terminal differentiation CD8^+^ T cells (% of terminal differentiation CD8^+^ T cells)	43.00 ± 20.33	46.77 ± 22.97	0.581
T cell differentiation subsets			
Tfh (% of CD4^+^ T cells)	20.17 ± 5.08	19.73 ± 5.91	0.798
Th1 (% of Th cells)	10.43 ± 4.64	8.64 ± 3.73	0.182
Th2 (% of Th cells)	18.25 ± 5.60	20.45 ± 5.88	0.227
Th17 (% of Th cells)	3.25 (2.27, 7.11)	2.63 (1.70, 3.55)	0.050
Tfh1 (% of Tfh cells)	11.70 ± 3.34	9.58 ± 2.33	0.024
Tfh2 (% of Tfh cells)	37.72 ± 6.57	42.72 ± 4.56	0.008
Tfh17 (% of Tfh cells)	6.52 ± 2.45	4.42 ± 1.82	0.004
Tc1 (% of Tc cells)	31.69 ± 13.02	33.81 ± 10.26	0.568
Tc2 (% of Tc cells)	17.20 (14.25, 21.65)	20.0 0 (15.18, 23.23)	0.865
Tc17 (% of Tc cells)	8.54 (5.17, 12.20)	9.31 (4.96, 11.18)	0.969
Th1/Th2	0.63 ± 0.33	0.45 ± 0.22	0.050
Th17/Th2	0.21 (0.17,0.33)	0.13 (0.09, 0.19)	0.003
Th1+Th17/Th2	0.90 ± 0.34	0.59 ± 0.25	0.002
Peripheral helper T cells (% of CD4^+^ T cells)	56.74 ± 5.23	58.00 ± 6.91	0.514
Activated Tfh (% of CD4^+^ T cells)	14.93 ± 4.12	14.65 ± 5.33	0.850
CD8+ T cells subsets			
Inhibitory CD8^+^ T cells (% of CD8 T cell)	21.09 ± 8.05	20.33 ± 7.34	0.754
Potential functional CD8^+^ T cells (% of CD8 T cell)	71.76 ± 10.22	73.50 ± 11.65	0.614
Total memory CD8^+^ T cells (% of CD8 T cell)	2.76 ± 1.35	3.50 ± 2.34	0.227
Homing memory CD8^+^ T cells (% of CD8 T cell)	72.70 ± 12.15	78.03 ± 12.28	0.170
Terminally senescent CD8^+^ T cells (% of CD8 T cell)	16.73 ± 8.83	17.22 ± 10.52	0.872
γδ T cells subsets			
γδ T cells (% of T cells)	5.25 ± 2.66	4.24 ± 1.77	0.162
Vδ 1^+^ (% of γδ T cells)	47.16 ± 22.88	54.96 ± 22.12	0.274
Vδ 2^+^ γδ T cells	50.36 ± 22.95	44.45 ± 22.26	0.408
Vδ 1^+^/Vδ 2^+^	0.70 (0.39, 1.77)	1.30 (0.42, 3.03)	0.251
NKG2D^+^Vδ 2^+^ (% of γδ T cells)	96.20 (86.85,98.50)	94.35 (87.50, 99.15)	0.948
PD1^+^ Vδ 2^+^ (% of γδ T cells)	5.45 (3.65,10.60)	5.87 (3.17, 11.78)	0.917
NKP30^+^Vδ 2^+^ (% of γδ T cells)	0.43 (0.13,0.92)	0.88 (0.43, 1.67)	0.074
NKP46^+^Vδ 2^+^ (% of γδ T cells)	1.76 (0.77,3.90)	1.41 (0.47, 2.92)	0.465
NKG2D^+^Vδ 1^+^ (% of γδ T cells)	58.87 ± 16.14	58.40 ± 11.86	0.917
PD1^+^Vδ 1^+^ (% of γδ T cells)	27.60 ± 13.06	25.15 ± 12.42	0.543
NKP30^+^Vδ 1^+^ (% of γδ T cells)	9.21 (3.18, 13.85)	10.25 (4.01, 15.05)	0.725
NKP46^+^Vδ 1^+^ (% of γδ T cells)	25.66 ± 17.05	18.34 ± 12.48	0.126

Tregs, regulatory T cells; Tfh, T follicle helper cell; Th, T helper; Tc, T cytotoxic; NKG2D, activated receptor of NK cells; NKP30, natural cytotoxicity triggering receptor 3; NKP46, natural cytotoxicity triggering receptor 1; PD1, programmed cell death protein 1. Data are presented as the mean ± standard deviation or a median (25–75% quartiles). A Student’s t test or Mann–Whitney U test was conducted. P < 0.05 was considered to indicate a statistically significant difference.

### Comparisons of NK Cell Subsets Between Groups

NK cell subsets in the peripheral blood were also measured. It was found that the percentage of early inhibitory NK cells was lower in the RIF group than that in the control group (*P* = 0.004; **Table 4**). No significant differences in the percentages of T, immature, mature, late inhibitory, activated, conventional killer, and virus-specific NK cells were observed between groups (all *P* > 0.05; **Table 4**).

**Table 4:** Comparisons of NK cell subsets between the RIF and control groups.

Cell type	RIF group (*n* = 21)	Control group (*n* = 20)	*P* value
NKT cells (% of lymphocyte)	7.89 ± 3.56	7.40 ± 2.92	0.629
Immature NK cells (% of NK cells)	60.20 (20.45, 86.45)	64.60 (33.93, 78.03)	0.774
Mature NK cells (% of NK cells)	37.90 (12.25, 79.60)	33.95 (20.33, 64.13)	0.648
Early inhibitory NK cells (% of NK cells)	41.79 ± 13.17	55.10 ± 14.62	0.004
Late inhibitory NK cells (% of NK cells)	6.44 (4.47, 10.65)	5.23 (2.21, 11.00)	0.335
Activated NK cells (% of NK cells)	45.12 ± 14.29	50.60 ± 16.92	0.269
Conventional NK cells (% of NK cells)	57.03 ± 23.35	52.38 ± 17.22	0.474
Viral specific NK cells (% of NK cells)	71.05 ± 16.24	61.68 ± 20.78	0.115

NK, natural killer; NKT, Natural killer T cell; RIF, recurrent implantation failure. The data are presented as the mean ± standard deviation or a median (25–75% quartiles). A Student’s t test or Mann–Whitney U test was conducted. P < 0.05 was considered to indicate a statistically significant difference.

Binary logistic regression analysis was performed for the 5 different indicators, and the results showed that Treg, Tfh17, and early inhibitory NK cells exhibited significant differences between the two groups (*P* < 0.05; **Table 5**).

**Table 5:** Logistic regression analysis of the effect of the five peripheral blood lymphocyte subsets on the RIF.

Cell type	*P* value	HR (95% CI)
Early inhibitory NK cells	0.023	0.900 (0.821–0.985)
Treg	0.018	0.246 (0.077–0.785)
Tfh1	0.775	1.076 (0.650–1.781)
Tfh2	0.124	0.836 (0.666–1.050)
Tfh17	0.044	1.730 (1.014–2.950)

HR, hazard ratio; CI, confidence interval; NK, natural killer; Tregs, regulatory T cells; Tfh, T follicle helper cell.

### Comparisons of Lymphocytes Between Groupsin Peripheral Blood Mononuclear Cells Between Groups

**Figure 1:**
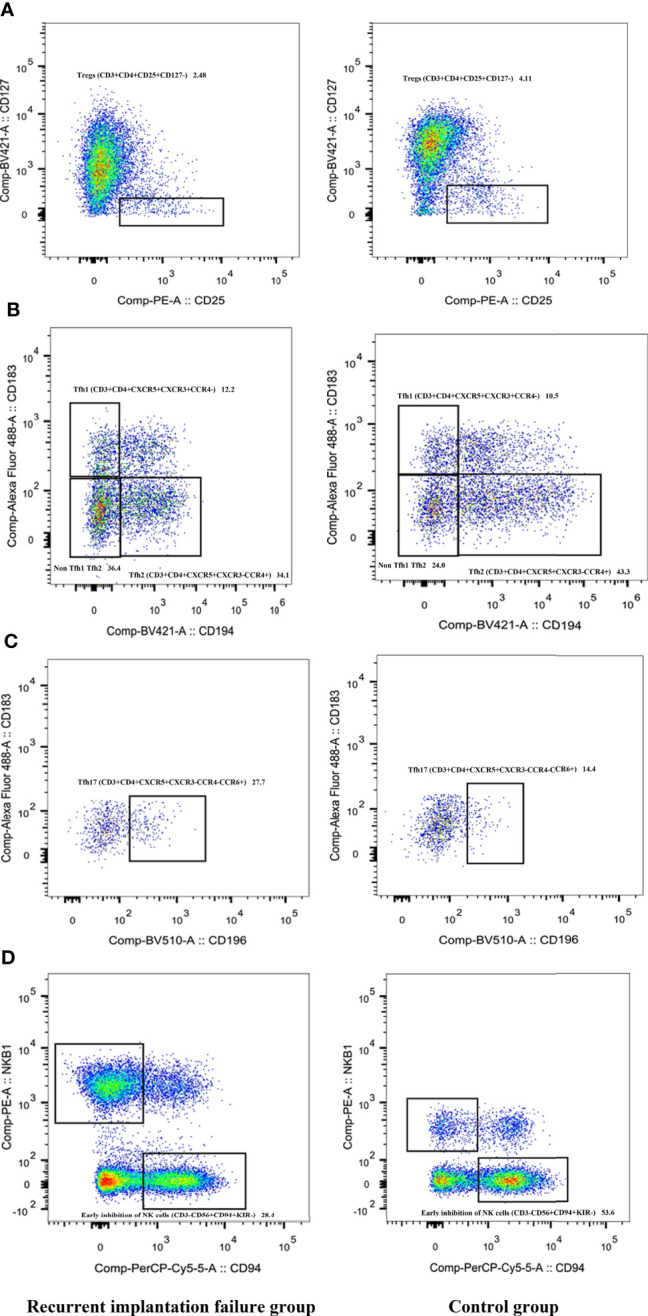
Comparison of Tregs **(A)**, Tfh1 **(B)**, Tfh2 **(B)**, Tfh17 **(C)**, and early inhibitory NK cells **(D)** between the RIF and control groups. RIF, recurrent implantation failure; NK, natural killer; Tregs, regulatory T; Tfh, T follicle helper.

To investigate the differences in peripheral blood lymphocytes between groups, their percentages in peripheral blood mononuclear cells were analyzed using flow cytometry. There were significant differences in the percentages of Tregs (CD3^+^CD4^+^CD25^+^CD127^-^), Tfh1 (CD3^+^CD4^+^CXCR5^+^CXCR3^+^CCR4^-^), Tfh2 (CD3^+^CD4^+^CXCR5^+^CXCR3-CCR4^+^), Tfh17 (CD3^+^CD4^+^CXCR5^+^CXCR3^-^CCR4^-^CCR6^+^), and early inhibitory NK cells (CD3-CD56^+^CD94^+^KIR^-^) between the two groups (**Figure 1**). The RIF group exhibited a significantly lower percentages of Tregs (2.24 ± 0.91 vs. 3.17 ± 1.09; *P* < 0.05; **Figure 1A**), a higher percentage of Tfh1 cells (11.70 ± 3.34 vs. 9.58 ± 2.33; *P* < 0.05; **Figure 1B**), a lower percentage of Tfh2 cells (37.72 ± 6.57 vs. 42.72 ± 4.56; *P* < 0.05; **Figure 1B**), a higher percentage of The Th17 cells (6.52 ± 2.45 vs. 4.42 ± 1.82; *P* < 0.05; **Figure 1C**), and a lower percentage of the early inhibitory NK cells (41.79 ± 13.17 vs. 55.10 ± 14.62; *P* < 0.05; **Figure 1D**), compared with the control group.

### Diagnostic Value of Biomarkers for RIF

**Figure 2:**
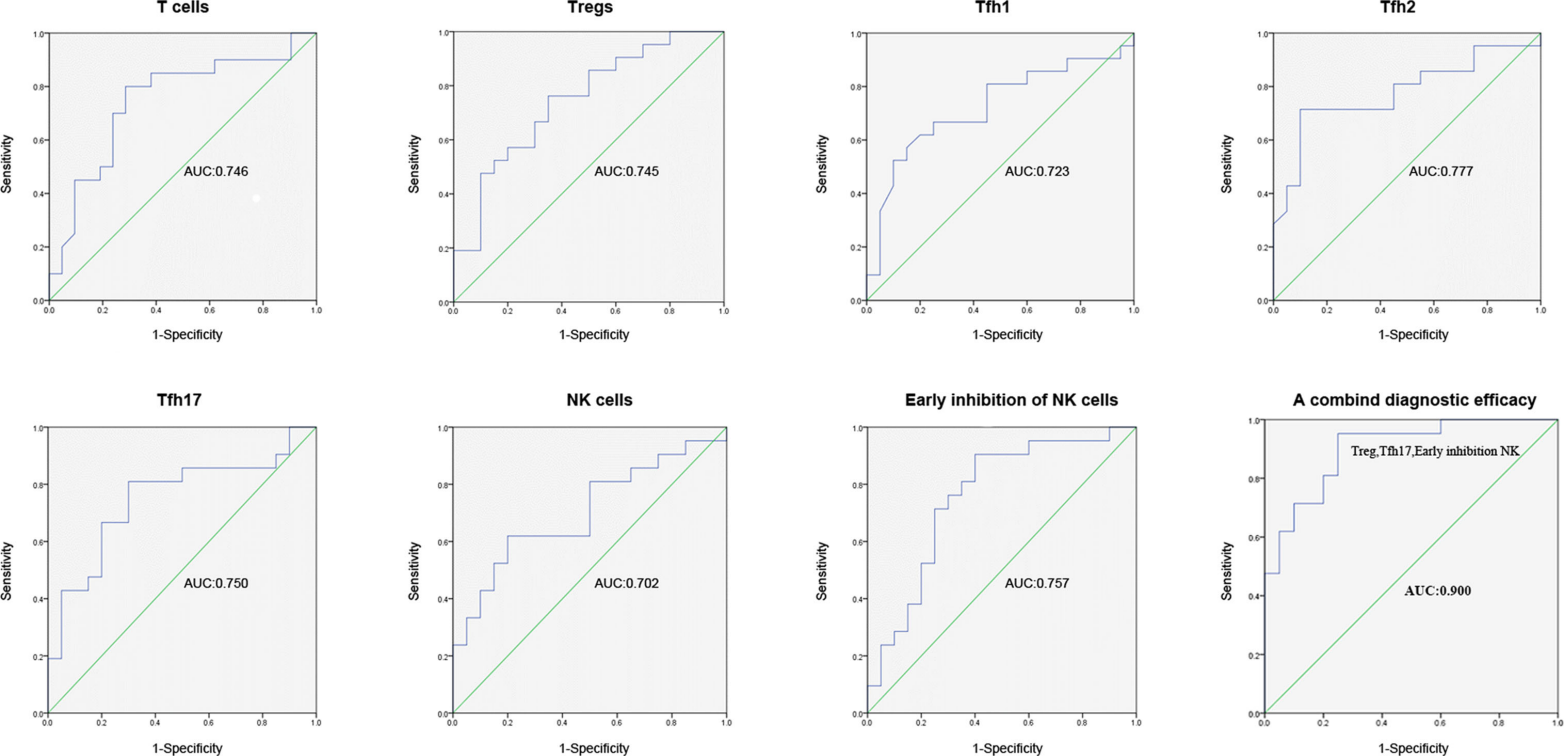
ROC curve for the efficiency of single (Tregs, Tfh1, Tfh2, Tfh17, and early inhibitory NK cells) and of combined biomarker diagnosis for RIF. ROC, receiver operating characteristic; RIF, recurrent implantation failure, NK, natural killer; Tregs, regulatory T; Tfh, T follicle helper.

In order to identify potential biomarkers for the diagnosis of RIF, logistic regression analysis and ROC curves were used to evaluate the diagnostic efficiency of single and combined biomarkers (**Figure 2**). The results showed that the AUCs of T cells, Tregs, Tfh1, Tfh2, Tfh17, NK cells, and early inhibitory NK cells were 0.746 (95% CI = 0.590–0.903, *P* = 0.007), 0.745 (95% CI = 0.595–0.896; *P* = 0.007), 0.723 (95% CI = 0.561–0.884; *P* = 0.015), 0.777 (95% CI = 0.629–0.926; *P* = 0.002), 0.750 (95% CI = 0.595–0.905; *P* = 0.006), 0.702 (95% CI = 0.540–0.864; *P* = 0.027), and 0.757 (95% CI = 0.604–0.910; *P* = 0.025), respectively. A combination diagnosis including all 3 markers revealed a significantly higher AUC of 0.900 (95% CI = 0.808–0.992; *P* < 0.001) than any marker alone. A combined diagnosis using these 3 markers had a high diagnostic value and may be able to distinguish the patients with RIF from patients with other conditions during ART.

## Discussion

Previous studies have focused on the aggregation of immune cells in endometrial tissue during the window of implantation, including NK cells, macrophages, dendritic cells, and Tregs, which provide a unique immune microenvironment for embryo implantation (15, 16). The immune cells found in the peripheral blood and endometrium are heterogeneous under different situations (17). We therefore assumed that there may be certain special biomarkers that may become abnormal under RIF. The detection of potential biomarkers to predict RIF in the peripheral blood would be preferable to endometrial biopsies. To the best of our knowledge, no reliable peripheral blood biomarker for RIF during the window of implantation has been reported yet.

In the present study, a systematic comparison of the subsets of peripheral blood lymphocytes, including T, CD4^+^ T, CD8^+^ T, NK, and γδ T cells in women with RIF (RIF group) and those with successful pregnancies (control group), was performed. The results showed that the RIF group exhibited a significantly higher percentage of Tfh1, Tfh17, and NK cells, compared with the control group. On the contrary, a significantly lower percentage of T cells, Tregs, Tfh2, and early inhibitory NK cells was identified in the RIF group, as compared with that in the control group. Finally, these findings indicated that a combined diagnosis using these 7 biomarkers (T cells, Tregs, Tfh1, Tfh2, Tfh17, NK cells, and early inhibitory NK cells) has a high diagnostic value and may be able to distinguish patients with RIF from other patients during ART. These findings suggested that the use of peripheral blood samples may be a safe and reliable potential diagnostic tool for women with RIF who underwent ART.

T cells could be divided into CD4^+^ and CD8^+^ T cells, according to their cell surface antigens (18). T cell subsets could be further differentiated into stem cell memory, central memory, effector memory, and effector T cells, which exist in peripheral tissues and blood, based on their effector memory differentiation. Those T cells can produce effector molecules upon activation (19). Our results indicated that there may be a decreased percentage of T cells in patients with RIF compared with fertile controls. A relationship between T cell activation and Th1 has previously been reported in women with RIF or RPLs (9). In addition, Li et al. further analyzed the levels of peripheral blood T cells in women with chronic endometritis (CE) and compared them with that in a non-CE group, in which patients had undergone recurrent miscarriage (RM) and RIF. However, no statistical difference was identified between the two groups (20), suggesting that peripheral blood T cells were not involved in the regulation of inflammatory responses in either RM or RIF. In the present study, flow cytometry was first used to investigate the expression of CD4^+^ and CD8^+^ T cell subsets for effector memory differentiation in women with RIF. However, no significant differences were observed between these subsets.

Of note, once the antigens were stimulated, naive CD4^+^ and CD8^+^ T cells could be characterized by several effector subsets based on their pattern of cytokine expression. These include type 1 T helper (Th1), Th2, Th17, Tregs, and T follicle helper cells (Tfh) for CD4^+^ T cells (21), as well as Tc1, Tc2, and Tc17 for CD8^+^ T cells (19), all of which play critical roles in maintaining immune tolerance. Tfh cells are generally considered the dominant T cell population, which could induce B cells to help reduce inflammation (22). The programmed cell death-1 molecule has been demonstrated to regulate the positioning and function of Tfh cells (23). A previous study also revealed an association between Tfh cells and human immunodeficiency virus (HIV) infection and showed that Tfh cells may play critical roles in antimicrobial defense, cancer, and autoimmunity (24, 25). A recent study found that E2 and P4 cooperate in the humoral immune response by favoring the expansion of different cyclic Tfh cell subsets (26). No study has yet reported the relationship between Tfh cells and RIF. To the best of our knowledge, the present findings were the first to suggest that Tfh1, Tfh2, and Tfh17 cells are associated with pregnancy outcomes in IVF treatment, and these types of cells may serve as novel indicators for the prediction of implantation success in patients undergoing ART.

Tregs that are distributed in peripheral blood circulation and tissues have been suggested to be necessary for the maintenance of maternal–fetal tolerance. An elevated expression of Tregs in the peripheral blood has been reported to be correlated with a favorable pregnancy outcome (27). It has also been shown that human chorionic gonadotropin could regulate the differentiation of Tregs in order to affect pregnancy outcomes in women with RIF (28). The results of certain studies focusing on human and murine models have revealed a reduction in the percentageof Tregs during RIF or unexplained infertility (29). A lower percentage of Tregs was also observed in the peripheral blood of women with RIF (30). In the present study, the levels of Tregs were significantly lower in patients with RIF, as compared with fertile controls, and these findings were consistent with previous studies. These findings provide a foundation for the use of Tregs for the detection of RIF.

NK cells constitute the dominant cell population in the endometrium, and they make contact with the extravillous trophoblast cells in the decidua during the early stage of pregnancy. Previous studies have focused on the role of NK cells in recurrent spontaneous abortion and RIF. The expression of NKP30 on cytotoxic NK cells (CD56dim CD16pos/neg) significantly increased in RIF (31). High NK cell numbers may be adisadvantage for ovarian reserve or function (32). Sacks et al. (33) reported that women with RIF had a higher NK cell activity in the peripheral blood, which was consistent with the findings of the present study. The present findings showed that patients with RIF may exhibit an increased number of NK cells. However, a study by Kolanska et al. (17) showed that peripheral blood NK cells alone were not able to reflect the risk of pregnancy failure or miscarriage, and it should therefore not be recommended for the management of RM and RIF. Nevertheless, differences in subsets of NK cells in the RIF and control groups were observed in the present study. The results showed a decrease in the percentage of early inhibitory NK cells in patients with RIF, which may provide some insights into the pathogenesis of RIF. To the best of our knowledge, no studies have investigated the early inhibition of NK cells in RIF to date. Further studies with larger samples need to be performed to re-verify these findings.

Pregnancy success or failure has been found to be correlated with the number of γδ T cells in the decidua of pregnant mice (34). Clark et al. (34) reported that γδ T cells could produce cytokines through an imbalance of Th1/2/3 cells in murine pregnancy decidua, leading to abortions. The present study focused on the relationship between γδ T cells in the peripheral blood and the success or failure of pregnancy, and the γδ T cells and subsets in the peripheral blood samples of patients with RIF were identified using flow cytometry. The results showed that there was no significant difference between groups. We therefore hypothesized that the γδ T cells in the peripheral blood and decidua were heterogeneous.

In conclusion, the present findings indicated that an increase in the percentage of Tfh1, Tfh17, and NK cells and a decrease in the percentages of Tregs, and T, Tfh2, and early inhibitory NK cells were associated with RIF. The data was strengthened by binary logistic regression modeling and the screening of three significant difference indicators: Treg, Tfh17, and early inhibitory NK cells. Combined diagnosis using these 3 molecular markers showed high diagnostic efficacy for assessing patients with RIF and could act as a novel potential biomarker for ART. We hope that our findings could provide a reference either for the clinical treatment of patients with RIF or for future large, well-designed studies.

## Data Availability Statement

The original contributions presented in the study are included in the article/**Supplementary Material**. Further inquiries can be directed to the corresponding author.

## Ethics Statement

The studies involving human participants were reviewed and approved by this research was carried out on patients from the Reproductive Medicine Center, Maternal and Child Health Hospital of Guangxi Zhuang Autonomous Region and approved by the Ethics Committee of the Maternal and Child Health Hospital of Guangxi Zhuang Autonomous Region for Reproductive Medicine. The patients/participants provided their written informed consent to participate in this study.

## Author Contributions

Designed the study: J-YCand HZ. Collected patients: Y-YT and X-HD. Performed the research: Y-JL, GL, and Y-QM. Statistical analyses: J-YC. Wrote the manuscript: J-YC. All authors contributed to the article and approved the submitted version.

## Funding

This research was supported by by the Natural Science Foundation of Guang Xi, China (2017GXNSFBA198150) to J-Y Cai, and the appropriate health technique development and Spreading and Application project of Guang Xi, China (S2020056) to H Zhou.

## Conflict of Interest

The authors declare that the research was conducted in the absence of any commercial or financial relationships that could be construed as a potential conflict of interest.

## Publisher’s Note

All claims expressed in this article are solely those of the authors and do not necessarily represent those of their affiliated organizations, or those of the publisher, the editors and the reviewers. Any product that may be evaluated in this article, or claim that may be made by its manufacturer, is not guaranteed or endorsed by the publisher.

## Supplementary Material

Supplementary Figure 1 Stepwise gating procedure for the flow cytometry analysis.
