# Supplementary figures and images for: Recurrent Implantation Failure May Be Identified by a Combination of Diagnostic Biomarkers: An Analysis of Peripheral Blood Lymphocyte Subsets
Source: Front Endocrinol (Lausanne). 2022 Jul 22;13:865807. doi: 10.3389/fendo.2022.865807 (PMC9353110; doi:10.3389/fendo.2022.865807)

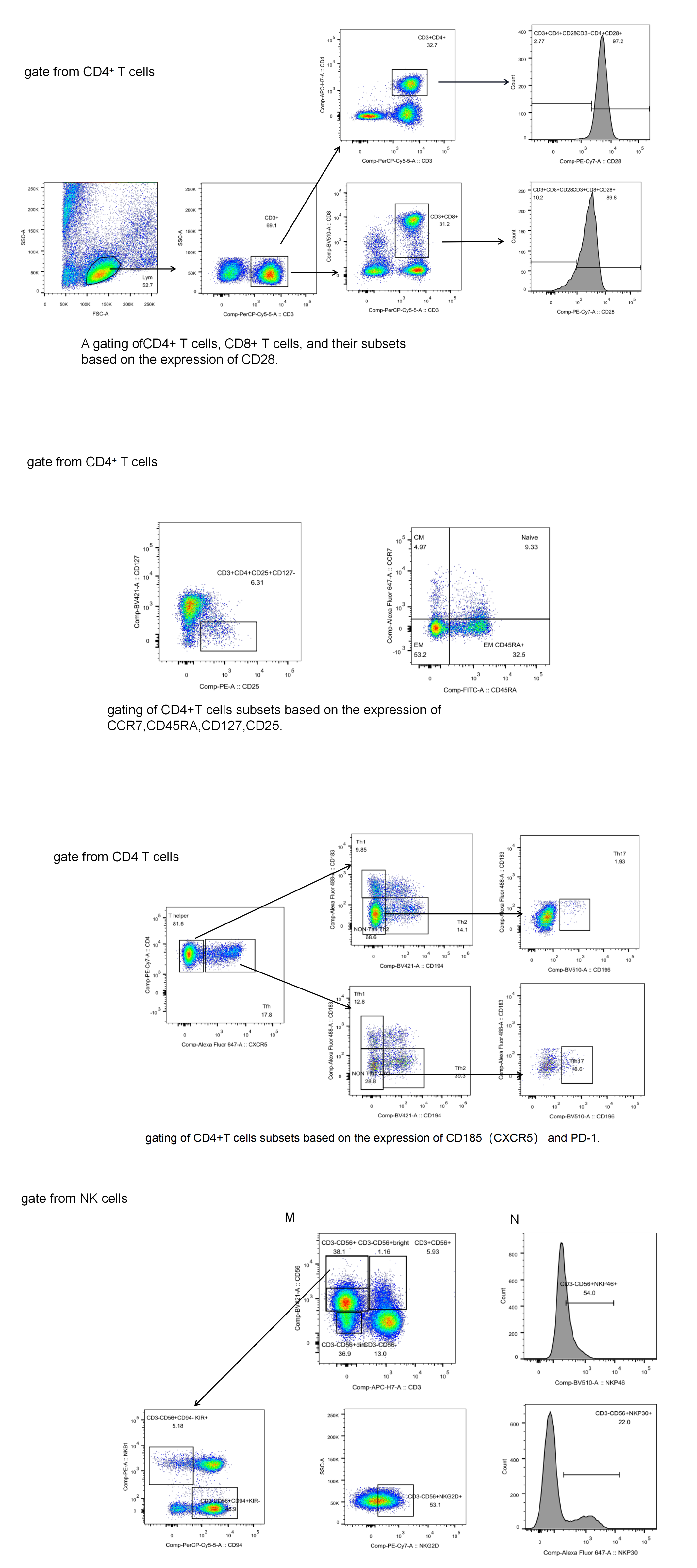

Supplement: Supplementary Figure 1 — Stepwise gating procedure for the flow cytometry analysis. [file Image_1.tif]
