# Supplementary material for: Recurrent Implantation Failure May Be Identified by a Combination of Diagnostic Biomarkers: An Analysis of Peripheral Blood Lymphocyte Subsets
Source: Front Endocrinol (Lausanne). 2022 Jul 22;13:865807. doi: 10.3389/fendo.2022.865807 (PMC9353110; doi:10.3389/fendo.2022.865807)
Supplement: Supplementary file 2 [file Table_1.docx]

**Supplementary Table S1** The classification for the ten-color antibody test for determination of the human immuno-phenotypes.

| **Cell type** | **Marker** | **Fluorochrome** |
| --- | --- | --- |
| T cells | CD3 | PerCP-Cy5.5 |
|  | CD4 | APC-H7 |
|  | CD8 | BV510 |
|  | CD28 | PE-Cy7 |
|  | CD127 | BV421 |
|  | CCR7 | AF647 |
|  | CD25 | PE |
|  | CD45RA | FITC |
| CD8^+^ T cells | CD3 | PerCP-Cy5.5 |
|  | CD28 | PE-Cy7 |
|  | CD57 | BV421 |
|  | CD38 | BV510 |
|  | PD-1 | AF647 |
|  | CD8 | FITC |
|  | HLA-DR | PE |
| NK cells | CD3 | APC-H7 |
|  | CD56 | BV421 |
|  | CD94 | PerCP-Cy5.5 |
|  | NKG2D | PE-Cy7 |
|  | P30 | AF647 |
|  | P46 | BV510 |
|  | NKB1（KIR） | PE |
| γδT cells | CD3 | APC-H7 |
|  | gd | BV421 |
|  | PD-1 | BB515 |
|  | P46 | BV510 |
|  | P30 | AF647 |
|  | NKG2D | PE-Cy7 |
|  | TCR Vd2 | PE |
| Th cells | CD3 | APC-H7 |
|  | CD4 | PE-Cy7 |
|  | CD8 | PerCP-Cy5.5 |
|  | CD183（CXCR3） | AF488 |
|  | CD196 (CCR6) | BV510 |
|  | CXCR5(CD185) | AF647 |
|  | CD194（CCR4） | BV421 |
|  | CD279 | PE |

APC-H7, allophycocyanin-cyanine H7tandem; FITC, fluorescein isothiocyanate; PE, phycoerythrin; PE-Cy7, phycoerythrin-cyanine 7 tandem; PerCP-Cy5.5, perdinin chlorophyll protein-cyanine 5.5 tandem; BV510, Brilliant Violet510; AF647, Alexa Flour 647.
