# Supplementary material for: Recurrent Implantation Failure May Be Identified by a Combination of Diagnostic Biomarkers: An Analysis of Peripheral Blood Lymphocyte Subsets
Source: Front Endocrinol (Lausanne). 2022 Jul 22;13:865807. doi: 10.3389/fendo.2022.865807 (PMC9353110; doi:10.3389/fendo.2022.865807)
Supplement: Supplementary file 3 [file Table_2.docx]

**Supplementary Table S2** Cell surface molecules observed in peripheral blood.

| **Cell type and subset** | **Antigen** |
| --- | --- |
| **Lymphocytes subsets** |  |
| T cell | CD3+ |
| NK cells | CD3-CD56+ |
| Helper T cell | CD3+CD4+ |
| Killer T cell | CD3+CD8+ |
| Double positive T lymphocytes | CD3+CD4+CD8+ |
| Th /Tc | CD3+CD4+/CD3+CD8+ |
| **T cell functional subsets** |  |
| Naïve CD4^+^T cells | CD3+CD4+CD45RA+CCR7+ |
| Terminal differentiated CD4^+^T cells | CD3+CD4+CD45RA+CCR7- |
| Central memory CD4^+^ T cells | CD3+CD4+CD45RA-CCR7+ |
| Effective memory CD4^+^ T cells | CD3+CD4+CD45RA-CCR7- |
| Exhaustion CD4^+^T cells | CD3+CD4+CD28- |
| Functional CD4^+^T cells | CD3+CD4+CD28+ |
| Regulatory T cells | CD3+CD4+CD25+CD127- |
| Naïve CD8^+^T cells | CD3+CD8+CCR7+CD45RA+ |
| Terminal differentiation CD8^+^T cells | CD3+CD8+CCR7-CD45RA+ |
| Central memory CD8^+^ T cells | CD3+CD8+CCR7+CD45RA- |
| Effective memory CD8^+^ T cells | CD3+CD8+CCR7-CD45RA- |
| Exhaustion of CD8^+^T cells | CD3+CD8+CD28- |
| Inactive specificity CD8^+^T cells | CD3+CD8+CCR7-CD45RA-CD127hi |
| Inactive specificity terminal differentiation CD8^+^T cells | CD3+CD8+CCR7-CD45RA+CD127hi |
| Persistent viral specificity CD8^+^Tcells | CD3+CD8+CCR7-CD45RA-CD127lo |
| Persistent viral specificity terminal differentiation CD8^+^T cells | CD3+CD8+CCR7-CD45RA+CD127lo |
| **T cell differentiation subsets** |  |
| Tfh | CD3+CD4+CXCR5+ |
| Th1 | CD3+CD4+CXCR5-CXCR3+CCR4- |
| Th2 | CD3+CD4+CXCR5-CXCR3-CCR4+ |
| Th17 | CD3+CD4+CXCR5-CXCR3+CCR4-CCR6+ |
| Tfh1 | CD3+CD4+CXCR5+CXCR3+CCR4- |
| Tfh2 | CD3+CD4+CXCR5+CXCR3-CCR4+ |
| Tfh17 | CD3+CD4+CXCR5+CXCR3-CCR4-CCR6+ |
| Tc1 | CD3+CD8+CXCR5-CXCR3+CCR4- |
| Tc2 | CD3+CD8+CXCR5-CXCR3-CCR4+ |
| Tc17 | CD3+CD8+CXCR5-CXCR3-CCR4-CCR6+ |
| Peripheral helper T cells | CD3+CD4+CXCR5-PD-1+ |
| Activated Tfh | CD3+CD4+CXCR5+PD-1+ |
| **CD8+T cells subsets** |  |
| Inhibitory CD8^+^T cells | CD3+CD8+PD-1+ |
| Potential functional CD8^+^T cells | CD3+CD8+CD28+ |
| Total memory CD8^+^T cells | CD3+CD8+CD28-CD57+ |
| Homing memory CD8^+^T cells | CD3+CD8+HLADR+ |
| Terminally senescent CD8^+^T cells | CD3+CD8+HLADR+CD38+ |
| **NK cell subsets** |  |
| NKT cells | CD3+CD56+ |
| Immature NK cells | CD3-CD56+hi |
| Mature NK cells | CD3-CD56+lo |
| Early inhibition of NK cells | CD3-CD56+CD94+KIR- |
| Late inhibitory NK cells | CD3-CD56+CD94-KIR+ |
| Activated NK cells | CD3-CD56+NKG2D+ |
| Conventional killer NK cells） | CD3-CD56+NKP30+ |
| Viral specificity killer NK cells | CD3-CD56+NKP46+ |
| **γδ T cells subsets** |  |
| γδ T cells | CD3+gd+ |
| Vδ 1+ | CD3+gd+Vδ2- |
| Vδ 2+ | CD3+gd+Vδ2+ |
| Vδ 1+/ Vδ 2+ |  |
| NKG2D+Vδ 2+ | CD3+gd+Vδ2+NKG2D+ |
| PD1+Vδ 2+ | CD3+gd+Vδ2+PD1+ |
| NKP30+Vδ 2+ | CD3+gd+Vδ2+NKP30+ |
| NKP46+Vδ 2+ | CD3+gd+Vδ2+NKP46+ |
| NKG2D+Vδ 1+ | CD3+gd+Vδ1+NKG2D+ |
| PD1+Vδ 1 + | CD3+gd+Vδ1+PD1+ |
| NKP30+Vδ1+ | CD3+gd+Vδ1+NKP30+ |
| NKP46+Vδ 1+ | CD3+gd+Vδ1+NKP46+ |

NK, natural killer; Tfh, T follicle helper cell; Th, T helper; Tc, T cytotoxic; NKT, Natural killer T cell; NKG2D, activated receptor of NK cells ; NKP30, natural cytotoxicity triggering receptor 3; NKP46, Natural Cytotoxicity Triggering Receptor 1; PD1, programmed cell death protein
